# 20% of US electricity from wind will have limited impacts on system efficiency and regional climate

**DOI:** 10.1038/s41598-019-57371-1

**Published:** 2020-01-17

**Authors:** S. C. Pryor, R. J. Barthelmie, T. J. Shepherd

**Affiliations:** 1 Department of Earth and Atmospheric Sciences, Cornell University, Ithaca, New York 14853 USA; 2 Sibley School of Mechanical and Aerospace Engineering, Cornell University, Ithaca, New York 14853 USA

**Keywords:** Atmospheric dynamics, Wind energy

## Abstract

Impacts from current and future wind turbine (WT) deployments necessary to achieve 20% electricity from wind are analyzed using high resolution numerical simulations over the eastern USA. Theoretical scenarios for future deployments are based on repowering (i.e. replacing with higher capacity WTs) thus avoiding competition for land. Simulations for the contemporary climate and current WT deployments exhibit good agreement with observed electricity generation efficiency (gross capacity factors (CF) from simulations = 45–48%, while net CF for WT installed in 2016 = 42.5%). Under the scenario of quadrupled installed capacity there is a small decrease in system-wide efficiency as indicated by annual mean CF. This difference is approximately equal to that from the two simulation years and may reflect saturation of the wind resource in some areas. WT modify the local near-surface climate in the grid cells where they are deployed. The simulated impact on near-surface climate properties at both the regional and local scales does not increase with increasing WT installed capacity. Climate impacts from WT are modest compared to regional changes induced by historical changes in land cover and to the global temperature perturbation induced by use of coal to generate an equivalent amount of electricity.

## Introduction

**Figure 1:**
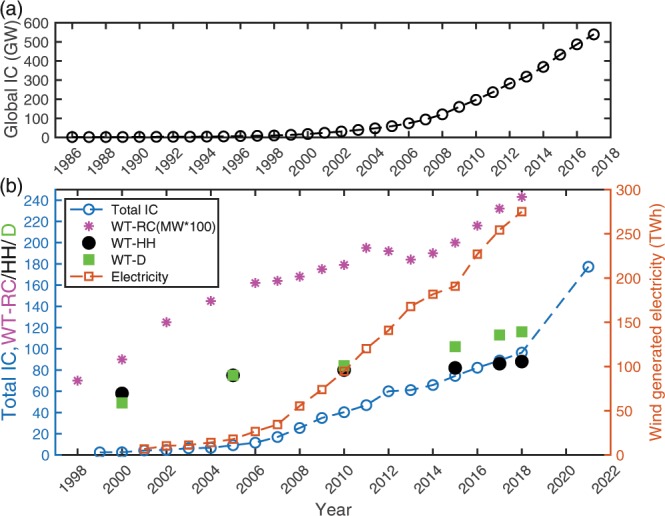
Total wind energy installed capacity (IC) in GW by year (**a**) globally^3^ and (**b**) in the USA^33^. Also shown in (**b**) is the projected WT installed capacity for 2021. Panel (b) also shows the average size of WT installed in that year (average rated capacity, WT-RC (in MW × 100, so 100 = 1 MW), hub-height (WT-HH, m) and rotor diameter (WT-D, m)) and the amount of electricity generated (TWh) from WT in the USA in each calendar year^33,64^.

**Figure 2:**
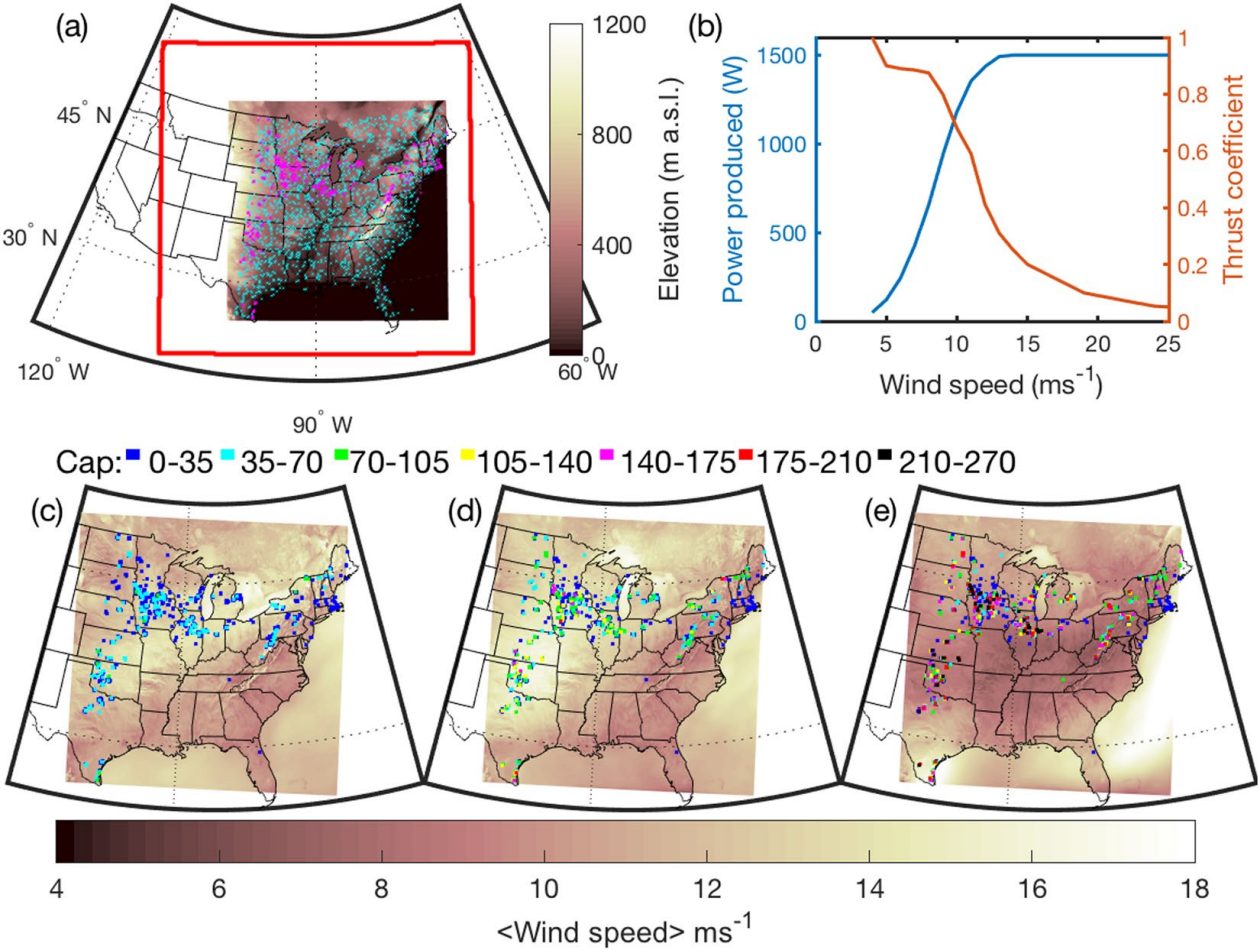
Simulation domain and the location and installed capacity of wind turbines (WT) deployed in each 4 × 4 km grid cell for the three scenarios. (**a**) Location of grid cells containing WT (magenta) as of the end of 2014 within the inner domain (d02) and the background grid cells (cyan) used in the statistical testing for regional effects. Red outline denotes the area covered by the outer domain (d01) and the shading denotes the terrain elevation. (**b**) Power (P) and thrust (c_t_) curves (i.e. dependence of power production and introduction of turbulence as a function of wind speed) for the most common WT in the 1 WT scenario (see Table 1 for a description of the other common WT types and those used in the repowering scenarios). Note values of P and c_t_ are only shown for wind speeds above cut-in (>4 ms^−1^). (**c**) Installed capacity (MW) in each 4 × 4 km grid cell for the 1 WT scenario, see legend titled ‘Cap’ and shown in the central row of the figure (a value of 100 MW per grid cell equates to a density of installed capacity of 6.25 MWkm^−2^). (**d**) As (**c**) but for the 2 WT scenario. (**e)** As (**c**) but for the 4 WT scenario. Background colors in (**c–e**) show the simulated mean wind speed in the third model layer (~mean WT HH for WT installed in 2014 (i.e. 1 WT)) from the noWT simulation in January, April and July 2008, respectively. Figure produced using MATLAB 2018a (https://www.mathworks.com).

The imperative to transform the energy system is well documented and great strides have already been made to increase the penetration of low carbon sources into the global electricity supply^1,2^. Over 340,000 wind turbines (WT) with a total capacity >591 GW were installed worldwide at the end of 2018 (ref. ^3^) with over 95 GW in the United States of America (USA) (ref. ^4^) (Fig. 1(a)). By 2015 WT supplied more than 4.3% of global electricity demand^5^. The power in the wind scales wit h the area swept by the WT blades and is thus proportional to the rotor diameter squared (D^2^). Wind speeds also generally increase with height. Thus, the total installed capacity (IC), average rated capacity and physical dimensions of WT being installed and wind generation of electricity have all exhibited marked growth in the USA over the last 20 years (Fig. 1(b)). The Central Plains exhibits the highest wind speeds and resource^6,7^ and thus the highest density of current WT installations (Fig. 2).

Wind generated electricity in the USA grew by 12% from 2016 to 2017 (from 227 to 254 TWh) and further increased to 275 TWh in 2018 (ref. ^4^) (Fig. 1(b)). Accordingly, WT provided 6.5% of total generation (4171 TWh, ref. ^8^) in 2018 (ref. ^4^). WT IC doubled between 2010 and 2017 and is projected to double again by 2021 (Fig. 1(b)). Quadrupling of installed WT capacity to meet a goal of 20% of USA electricity supply by 2030 (ref. ^9^) falls well below theoretical limits for potential wind generated electricity^10,11^, but implies approximately a further doubling of installed capacity after 2021.

Here we address whether the expansion of WT IC in the eastern USA is likely to lead to either: (i) Decreased overall efficiency of electrical power production from the WT fleet^10^. (ii) Unacceptably large inadvertent modification of climate at the local or regional scale^12^. This is the first study to address these questions and employs a traceable and transparent experimental design in which the increase in IC is achieved by replacement of WT with newer, higher capacity WT.

Impacts on near-surface climate properties adjacent to operating WT are inevitable given that they harvest kinetic energy and in doing so increase turbulence intensity and mixing in the lower atmospheric boundary layer^13–15^. WT thrust coefficients that determine momentum extraction and introduction of turbulence are non-linear functions of wind speeds (Fig. 2(b)). Accordingly, local and downstream effects from WT are a complex function of atmospheric conditions and WT characteristics (thrust curve, WT hub-height (HH) and rotor diameter (D)).

Upstream WT and WT arrays reduce inflow wind speeds to downstream WT and wind farms. This reduces system-wide efficiency of electrical power production and is more pronounced in high density, large WT arrays^10,16^. Limits on horizontal and vertical fluxes of kinetic energy to replace that removed by WT place a fundamental limit on the amount of energy WT can extract per unit surface area^10^. Theoretical research investigating this issue has invoked infinite wind farms and/or assumed large areas of high installed capacity density that exceed current levels (Fig. 2(c)). One such analysis conducted for June–September 2001 assumed a WT array deployed over a 10^5^ km^2^ region centered on Kansas and installed capacities of 0.3–100 MWkm^−2^. The results indicate marked overall system-wide inefficiencies with increasing IC and that large-scale electricity generation rates from massive arrays are limited to approximately 1.0 Wm^−2^ (ref. ^10^).

Early modeling research designed to examine downstream climate effects from WT arrays essentially treated them as large roughness blocks^17–19^. However, WT arrays are porous (WT occupy <2% of the wind farm area^20^) and do not perturb the atmosphere in a manner analogous to a large roughness block^21^. The advent of wind farm parameterizations that represent aspects of WT aerodynamics^21,22^ mean it is now possible to simulate the action of WT on the atmosphere more realistically. Limited previous studies have utilized these wind farm parameterizations and have found smaller impacts on local and regional climate. However, they have employed either relatively coarse discretization^23,24^, short simulation periods^25^, small simulation domains^10,25^ and, with exceptions^15^, have used idealized assumptions about the locations and/or characteristics of the WTs.

For the reasons described above, questions remain about the impact of WT deployments on system-wide efficiency of electrical power production and regional climates (i.e. time averaged statistical properties over large spatially-coherent areas), and how these effects may scale in response to the changes in the WT fleet necessary to achieve the goal of 20% of US electricity from wind by 2030 (ref. ^9^). The simulations presented are the first to be conducted at convection permitting resolution over a sufficiently large domain (Fig. 2(a)) and long duration to capture possible regional scale impacts and characterize the entire seasonal cycle. These simulations are conducted for a year with relatively high wind speeds (2008) and one with lower wind speeds (2015/6). The interannual variability of wind resources over the USA is a function of internal climate modes^26–28^, and these two years are selected to sample differing phases of the El Niño-Southern Oscillation (ENSO) and thus provide a first estimate of the influence of internal climate variability on system efficiency and climate impacts from WT.

The outer domain (d01) used in the simulations comprises 319 × 319 grid cells of 12 km × 12 km, and the inner domain (d02) comprises 675 × 675 grid cells (i.e. 455,625 grid cells in total) of 4 km × 4 km (Fig. 2(a)). Simulations are conducted for:

(i) Conditions without any wind turbines (noWT).
(ii) Explicit WT geographic locations, HH, D, thrust and power curves for each WT installed as of the end of 2014 (in a scenario called 1 WT, Fig. 2(c)).
(iii) A scenario in which the WT installed capacity is doubled (2 WT, Fig. 2(d)).
(iv) A scenario in which the WT installed capacity is quadrupled (4 WT, Fig. 2(e)).

The installed wind energy capacity in d02 for the 1 WT scenario is ~31.3 GW (Fig. 2(c)). This equates to almost half (48%) of the total national installed capacity at the end of 2014. For simulations of 1 WT all >18,200 operating WT in d02 were georeferenced and more than 98% are characterized using 28 WT-specific power and thrust curves, HH and D (Table 1) for use in a wind farm parameterization to compute WT power production and flow disturbance^21^. Fewer than 2% had missing information and/or were types present in small numbers. These were allocated to one of two synthetic WT characteristic definitions.

**Table 1:** The 8 most common types of wind turbines deployed in eastern USA as of the end of 2014.

Manufacturer and model	Hub-height (HH) (m)	Rotor diameter (D) (m)	Power (P) and/or thrust (c_t_) curves	Rated capacity (RC) (MW)	Number deployed in simulation domain
WT specifications for the top 8 most common WT types as installed at the end of 2014.					
1. GE 1.5 SLE	80	77	Explicit	1.5	4417
2. Vestas V82	80	82	Explicit	1.65	1730
3. GE 1.5 XLE	80	82.5	P, c_t_ from GE 1.5 SLE	1.5	1242
4. Nordex N117	91	117	Scale P from Nord2500, c_t_ average from WT with equal RC	2.4	1058
5. Gamesa G87	78	87	P, c_t_ average of WT with equal RC	2	953
6. GE 1.6_82.5	80	82.5	P, c_t_ scaled from GE 1.7 MW	1.6	921
7. Siemens 2.3_101	80	101	Taken from analogous WT	2.3	696
8. GE 1.5S	64.7	70.5	P from GE 1.5 SLE, c_t_ explicit	1.5	602
**WT used in scenarios**					
Manufacturer and model	Hub-height (HH) (m)	Rotor diameter (D) (m)	Power (P) and/or thrust (c_t_) curves	Rated capacity (RC) (MW)	Source of power/thrust curves
Vestas 3 MW	80	90	Explicit	3	Confidential
NREL 5.2 MW	90	126	Explicit	5.2	^65^
LW Demonstration machine	110	164	Explicit	8.2	^66^

Also shown are the turbine descriptive parameters: Hub-heights (HH), rotor diameters (D) and the origins of the power and thrust curves (see examples in Fig. 2b) used in wind farm parameterization. Where explicit machine specific power and/or thrust curves were not available values are scaled from similar WT (as noted in the table). The lowest three rows show comparable information for the WT used in the repowering scenarios. Where thrust coefficients are not available, the average c_t_ curve shown in Table S1 is applied.

Two theoretical repowering scenarios are used to represent near-term doubling (2 WT) and quadrupling (4 WT) of the WT installed capacity. The repowering *scenarios* presented are defined in a manner consistent with use of scenarios by the Intergovernmental Panel for Climate Change (IPCC), the International Energy Agency (IEA) and the Organization for Economic Cooperation and Development. They ‘are neither predictions nor forecasts, but are useful to provide a view of the implications of developments and actions’ (ref. ^29^). In these scenarios the number of WT is held constant but older generation WT are replaced by newer, higher capacity WT (in a process known as repowering). For the doubling scenario (2 WT), all WT that have a rated capacity <2.1 MW are replaced with a 3 MW WT, while those with a rated capacity >2.1 MW are replaced with a 5.2 MW WT yielding a total installed capacity of 61.7 GW (i.e. 1.97 times the installed capacity as of the end of 2014, and referred to as 2 WT, Fig. 2(d)). The quadrupling scenario (4 WT) replaces 3 MW WT in the doubling scenario with 8.2 MW WT and holds constant the 5.2 MW WT from the doubling scenario (Fig. 2(e)).

Not all future increases in IC will be achieved via repowering, but use of repowering scenarios avoids speculation regarding where new wind turbine arrays will be developed. Both the Global Wind Energy Council^30^ and the IEA^31^ include “substantial and increased repowering” into their growth scenarios, and indicate repowering “becoming a significant factor after 2025” (ref. ^30^). Thirty-percent of US wind farms are expected to undergo repowering by the end of 2020 (ref. ^32^), and a total of 3.6 GW of repowering projects were completed in 2017 and 2018 (ref. ^33^).

A survey of published studies reports average installed capacity densities (ICD) for onshore wind turbine arrays of 7.1 to 11.5 MWkm^−2^ (refs. ^34–37^). That study also relates evidence that the mean (and range) ICD in Europe is actually 19.8 (6.2–46.9) MWkm^−2^ and outside Europe is 20.5 (16.5–48) MWkm^−2^ (ref. ^38^). ICD from offshore wind farms in Europe are generally lower than those onshore^38^, but those with project areas of less than 16 km^2^ (i.e. the size of the grid cells in the current study) have an average of 16 MWkm^−2^ (ref. ^39^). These empirical ICD are used to constrain WT installed capacity in the repowering scenarios to ≤16.25 MWkm^−2^ (i.e. 260 MW in a 16 km^2^ grid cell). For the majority of grid cells containing wind turbines the ICD is <8 MWkm^−2^, even in the quadrupling scenario. Only 15 (<<1%) of the 455,625 grid cells in the quadrupling scenario exceed a total installed capacity of 200 MW (i.e. 12.5 MWkm^−2^) and none exceed 260 MW (i.e. 16.25 MWkm^−2^) (Fig. 2(e)).

System-wide electrical power production from the resulting WT fleets are described using total annual electricity production (as derived from the wind farm parameterization) and gross capacity factors (CF). Gross CF are the ratio of electrical power produced according to the wind farm parameterization to the maximum possible production determined by the cumulative rated capacity of WT in that scenario (1 WT, 2 WT or 4 WT).

The impact of WT on near-surface climate variables is assessed by computing mean pairwise differences in model output (XWT-noWT, where X = 1, 2 or 4) in each grid cell. The results are presented; (i) for all individual grid cells, (ii) as a function of distance from the closest WT and (iii) aggregated to the regional level. We also quantify the impact of WT operation on different percentiles of near-surface air temperature (T2M) probability distribution and by hour of the day.

## Results

### System efficiencies of electrical power production for the WT scenarios

**Figure 3:**
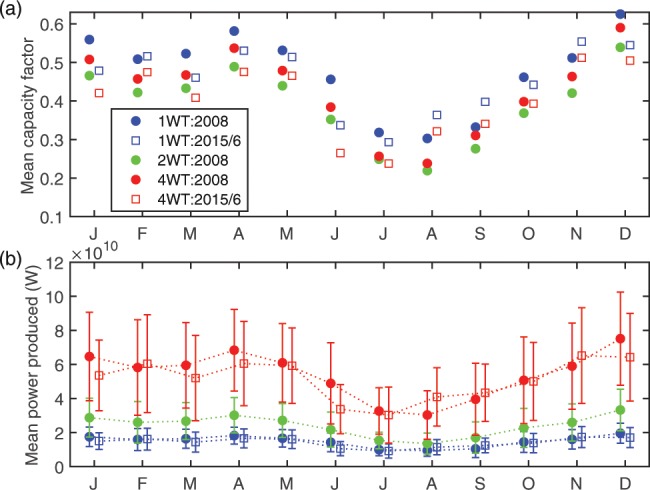
Mean monthly WT (**a**) gross capacity factors and (**b**) power production based on 10-minute simulated values for all calendar months (J = January, F = February etc) of 2008 and 2015/2016 for the three WT deployment scenarios (1 WT, 2 WT and 4 WT). Vertical bars in (**b**) denote ± 1 standard deviation around the mean value computed using the 10-minute power output time series for cumulative power output from all WT in the study domain (d02).

Consistent with evidence that the negative ENSO phase is typically associated with significantly stronger lower-troposphere wind speeds over the eastern USA^26–28^, total annual electricity production in the 1 WT simulation for the climates of 2008 and 2015/6 are 131 and 124 TWh, respectively. Accordingly, the annual mean gross CF for the 1 WT scenario is 48% for 2008 when La Niña conditions prevailed and 45% for 2015/6 when El Niño conditions occurred (Fig. 3).

Due to the seasonality of wind speeds (Fig. 2(c–e)), electrical power production from WT and gross CF also exhibit marked intra-annual variability. For example, gross CF for the 1 WT simulation of 2008 are 56% in January, 58% in April and 32% in July, while gross CF for the 1 WT simulation for January 2016, April 2015 and July 2015 are 48%, 53% and 29%, respectively (Fig. 3).

Gross CF reflect an upper bound on possible electrical power production and are higher than observed net CF for WT operating in the USA (∼36% for long-term operating wind farms in the d02 domain^27^ and 42.5% for WT installations commissioned in 2016 (ref. ^40^)). They assume 100% WT availability and performance (i.e. no downtime for maintenance or curtailment of production, no electrical or turbine losses) and neglect the WT wake interactions within a grid cell. The discrepancy between simulated gross CF and observed net CF of approx. 9 to 12% is consistent with empirical estimates of power production losses from these sources. Observed levels of curtailment over the study domain were generally ≤4% during 2007–2012 (ref. ^27^). Availability of WT deployed onshore tends to decrease with WT age^41^ but is typically ≥98% (ref. ^42^). Total observed wake losses within land-based WT arrays are estimated to decrease power production and CF by ≤5% (ref. ^43^).

Total annual electricity production generated in the three WT scenarios (1 WT, 2 WT and 4 WT) for the 2008 climate are; 131, 211 and 474 TWh, respectively. Equivalent numbers for the 1 WT and 4 WT scenarios in 2015/6 are 124 and 449 TWh. Power production and gross CF thus indicate a reduction in overall system-wide efficiency of electrical power production in the repowering scenarios. For example, quadrupling IC by repowering increases gross total annual electricity production in the two simulation years by a factor of ~3.63. Gross CF are lowest for the 2 WT scenario (Fig. 3). This is likely because repowering from 1 WT to 2 WT involves replacement of older WT with WT of higher generation capacity but with relatively similar HH (see Table 1). The 3 MW WT used in the 2 WT scenario has a HH of 80 m which is ~ mean HH in WT installed at the end of 2014 (of 83 m), and is the HH of 47% of new installations in 2017 (ref. ^4^). The quadrupling scenario relies on deploying WT with considerably larger RC and D, and modestly higher HH, consistent with the historical tendencies (Fig. 1(b)). Use of these WT leads to higher gross CF (Fig. 3). The reduction in mean gross CF for the 4 WT scenario relative to the 1 WT scenario is 5% in both years and is thus similar to the difference in annual gross CF in 2008 versus 2015/6 (∼3%). Thus, although some individual grid cells in the 4 WT scenario are likely close to the limit at which maximum kinetic energy extraction occurs^10^, system-wide power production and CF remain relatively high even for an extreme scenario in which quadrupling of installed capacity is achieved solely by repowering.

### Climate impacts from wind turbines

**Figure 4:**
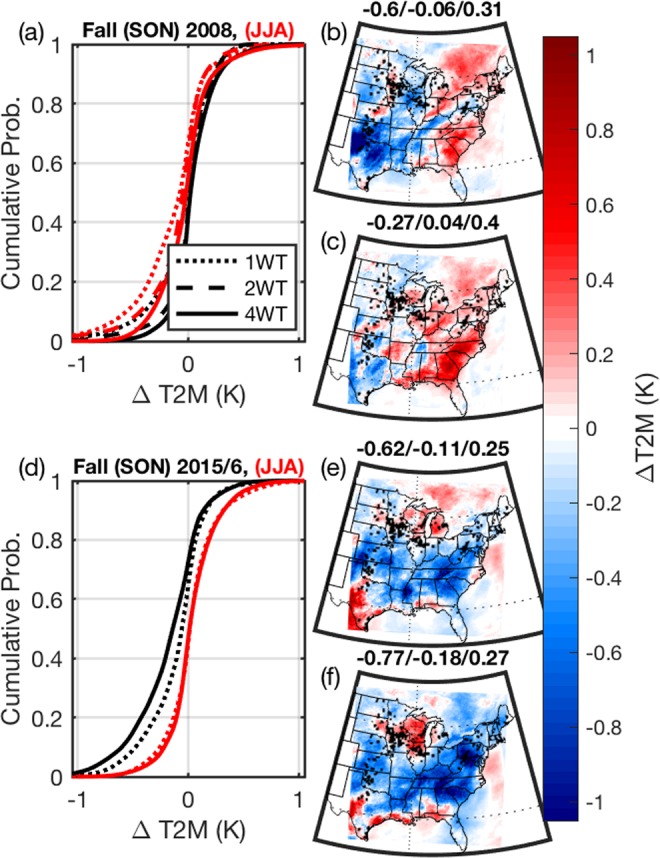
Impact of WT on near-surface (2-m) air temperatures (T2M). (**a,d**) Cumulative probability density of all >400,000 grid cell mean pairwise differences (XWT minus noWT of hourly T2M) during fall (SON, black lines) and summer (JJA, red lines) of 2008 and 2015/6 for the three WT scenarios; 1 WT, 2 WT (2008, only) and 4 WT. (**b,c)** Spatial fields of mean pairwise difference in SON 2008 T2M for 1 WT and 4 WT, respectively. (**e,f)** show the same but for SON 2015/6. Values above panels (b,c,e,f) denote the 5^th^ percentile/50^th^ percentile/95^th^ percentile values grid cell mean pairwise differences. The black + symbols in these panels show grid cells containing WT. Figure produced using MATLAB 2018a (https://www.mathworks.com).

**Figure 5:**
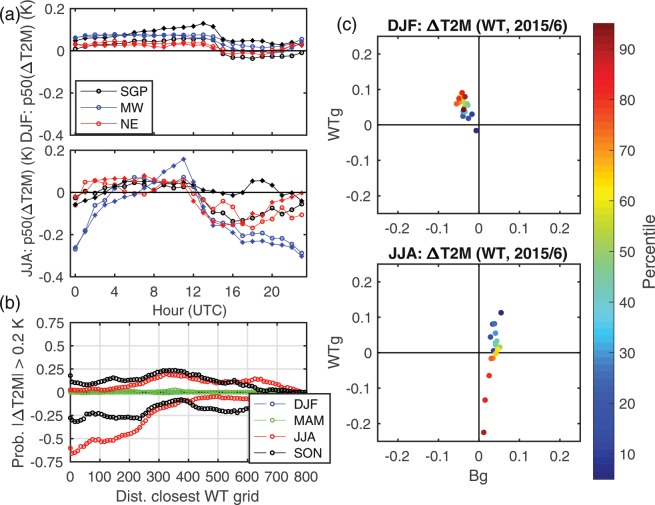
Impact of WT on near-surface (2-m) air temperatures (T2M). (**a)** Diurnal cycle of median wintertime (DJF) and summertime (JJA) air temperature perturbation (1 WT minus noWT) for all WT grid cells in three sub domains; Southern Great Plains (SGP, latitudes below 33.3°N, all longitudes west of 96.5°W), Midwest (MW, latitude: 42–45°N, longitude: 96–90°W), Northeast (NE, latitude: 42–50°N, all longitudes east of 87°W), for 2008 (circles) and 2015/6 (diamonds). (**b**) Probability of a mean T2M perturbation >0.2 K for each climatological season of 2008 for 4 WT minus noWT as a function of distance (km) from the closest grid cell containing WT. The probability that a given cell at that distance has a perturbation of >+0.2 K is shown as positive, while the probability that a given cell at that distance exhibits cooling >0.2 K is shown as negative. E.g. In summer a grid cell 100 km from the closest WT has a 50% chance of having ΔT2M <−0.2 K, and a 5% chance of ΔT2M >0.2 K. (**c**) Scatterplots of 1 WT mean ΔT2M for winter (DJF) and summer (JJA) of 2015/6 expressed as a function of the decile in the T2M probability distribution in the noWT simulation for 1800 remote (Bg) and 1800 WT (WTg) grid cells.

Climate is defined as the long-term mean averaged over a spatial extent that may be local, regional or global. In the context of the Intergovernmental Panel for Climate Change (IPCC) local-scale typically refers to physical dimensions of a few kilometers, while regional scale is used to represent tens to hundreds of kilometers^44^. The *a priori* expectation is that if the WT fleet substantially impacts regional climate over the eastern USA, this should be manifest as large-scale spatially coherent regions of mean grid-cell specific pairwise differences ($\frac{1}{n}\mathop{\sum }\limits_{i=1}^{n}\,XW{T}_{i}-W{T}_{i}$, where i = 1 to the total number of hours in a season (n), and X = 1, 2 or 4). However, maps of the mean grid cell specific pairwise differences for all climate parameters indicate complex patterns of varying sign. Near-surface air temperature (T2M) difference fields for fall (SON) 2008 and fall 2015 (Fig. 4) illustrate two important features of these patterns: There are marked variations in the sign (and magnitude) of T2M differences even in areas of dense WT developments (e.g. Texas versus Iowa, Fig. 4(b)) consistent with a key role of local climate (and surface energy partitioning) in determining WT impacts. There is also large intra-annual and inter-annual variability in the impacts of WT on near-surface climate due to variations in the base climate. T2M response to WT in fall 2015 exhibits the opposite sign to that from 2008 with, for example, cooling (versus warming) of mean T2M over the southeastern USA irrespective of the WT scenario (cf. Fig. 4(b,c,e,f)). The average diurnal profiles of T2M differences (1 WT minus noWT) from WT grid cells in three sub-regions of the model domain (the Southern Great Plains (SGP), Midwest (MW) and Northeast (NE)) indicate the expected variations with season and hour of the day; with warming of the nighttime hours in both winter and summer in all regions, but cooling of the daylight hours during summer (Fig. 5(a)). The warming of T2M during winter-time nighttime hours particularly in the Midwest is consistent with analysis of skin temperatures from satellites^14^. The median values of T2M differences from all WT grid cells in the Southern Great Plains (≈+0.1 K) which is smaller than the value for wind farms in west central Texas derived from MODIS (≈+0.5 K) likely due to differences in spatial averaging (MODIS data were used at 1.1 km resolution) and exclusion of cloudy-pixels in the MODIS processing algorithm for skin temperature. Data from a similar satellite-based study over five wind farms in Iowa (i.e. in the Midwest) indicated mean perturbations of nighttime (10:30 pm, local time) skin temperature (2010–2013 minus 2003–2007) of +0.12–0.44 K during cloud-free conditions^45^. Output from the WRF simulations indicate that during that hour (0400-0500) the median T2M perturbation due to WT is positive (i.e. near-surface warming) for the climate of 2008 but negative (i.e. cooling) for the summer of 2015/6. Thus, consistent with experimental research^45^, this analysis of the WRF output further emphasizes important inter-annual variability in local air temperature impacts from WT operation. This variability and the strong regional variations in WT impacts on near-surface air temperatures arise in part from differences in the surface energy balance. They critically depend on the magnitude of net radiation and soil moisture availability (and thus seasonal precipitation). For example, simulated (and observed) precipitation over much of the southern Great Plains was higher in 2015/6 than 2008 (see SI Figs. 2–4), resulting in marked difference in the WT-induced climate perturbations over the SGP (Fig. 5(a)). The implication is that to accurately characterize the net regional climate impact from WT operation there is a need to sample (experimentally or numerically) a range of climate states (i.e. sample internal climate variability) and investigate the effects over large spatial domain. The further inference is that the mean impact from WT installations even on local climate are likely to be regionally specific.

**Figure 6:**
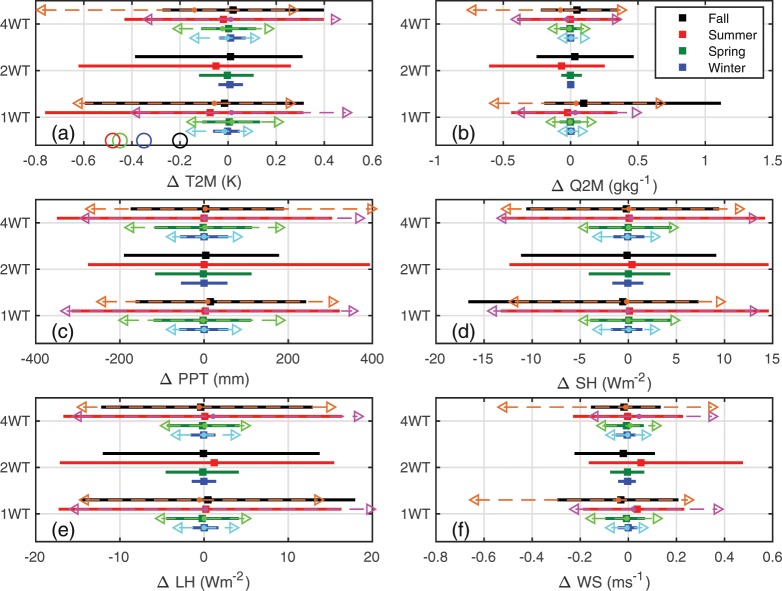
Median (50^th^ percentile) and 5^th^ to 95^th^ percentile span of grid cell mean pairwise hourly differences (Δ computed as XWT minus noWT) in (**a**) air temperature at 2-m (T2M), (**b**) specific humidity at 2-m (Q2M), (**d**) sensible heat flux (SH), (**e**) latent heat flux (LH) and (**f**) and wind speed at 10-m (WS) for the three WT scenarios (1 WT, 2 WT, 4 WT). Panel (c) shows the difference in total seasonal precipitation (PPT). Squares and solid bars denote results for 2008, while circles and dashed lines headed with triangles that are shown in the lighter shades of each color indicate results for March 2015 – February 2016. Results for the four climatological seasons in each WT scenario are slightly vertically displaced to aid legibility. Open circles on the x-axis of frame (**a**) denote mean changes in T2M over the US Central Plains region estimated due to land use land cover change over the twentieth century^46^.

When integrated across space WT-induced perturbations of near-surface properties average to close to zero. For example, median values of all >400,000 grid cell specific mean pairwise differences (XWT minus noWT) in T2M lie between −0.14 and 0.02 K (depending on season and WT scenario, Fig. 6(a)). Comparable values for the difference in specific humidity at 2-m (Q2M), total seasonal precipitation (PPT), sensible and latent heat fluxes (SH and LH) and wind speed at 10-m (WS) are; −0.08 to 0.10 gkg^−1^, −2.8 to 15 mm, −0.58 to 0.39 Wm^−2^, −0.57 to 1.20 Wm^−2^ and −0.04 to 0.05 ms^−1^ (Fig. 6(b–f)). Thus, even large-scale expansion in WT installed capacity does not appear to greatly impact near-surface climate at the regional scale. There are, as described above, impacts at the local scale (i.e. in grid cells containing WT) that, for T2M, are of similar magnitude to experimental data^13–15^. Maximum cooling in WT grid cells occurs in summer (Fig. 5(a,c)), as a result of enhanced mixing of hotter air away from the surface during the daylight hours. The highest deciles (i.e. top 20%) of T2M values (i.e. daytime peak air temperatures) in WT grid cells are decreased by an average of 0.2 K due to the additional mixing resulting from WT operation (Fig. 5(c)). Conversely, largest local warming occurs in winter (Fig. 5(a,c)). T2M during DJF is on average warmed by upto 0.1 K across all deciles due to the mixing down of warmer air from aloft by the WT. This effect is absent in the lowest decile, in part because the coldest nighttime hours in the simulations presented here are typically associated with lower than average wind speeds near typical WT HH. These effects on near-surface climate are smaller in background grid cells (i.e. grid cells displaced from WT, Fig. 5(c)) and diminish with increasing distance from the grid cells in which WT are deployed (Fig. 5(b)). The impacts on T2M are also reduced in the 4 WT scenario relative to the 1 WT scenario (Fig. 6(a)) likely because of the higher WT HH used in the 4 WT scenario.

To provide a context for the local T2M perturbations from WT, area averaged perturbations in near-surface air temperatures due to changes in land use – land cover from preindustrial to end of twentieth century are ~ −0.2 to −0.5 K (depending on climatological season, Fig. 6(a))^46^. In our simulations, T2M perturbations from WT do not exceed those from LULC change except at the local scale (i.e. in individual 4 km × 4 km grid cells containing WT) during the summer of 2008 for the 1 WT simulation.

**Figure 7:**
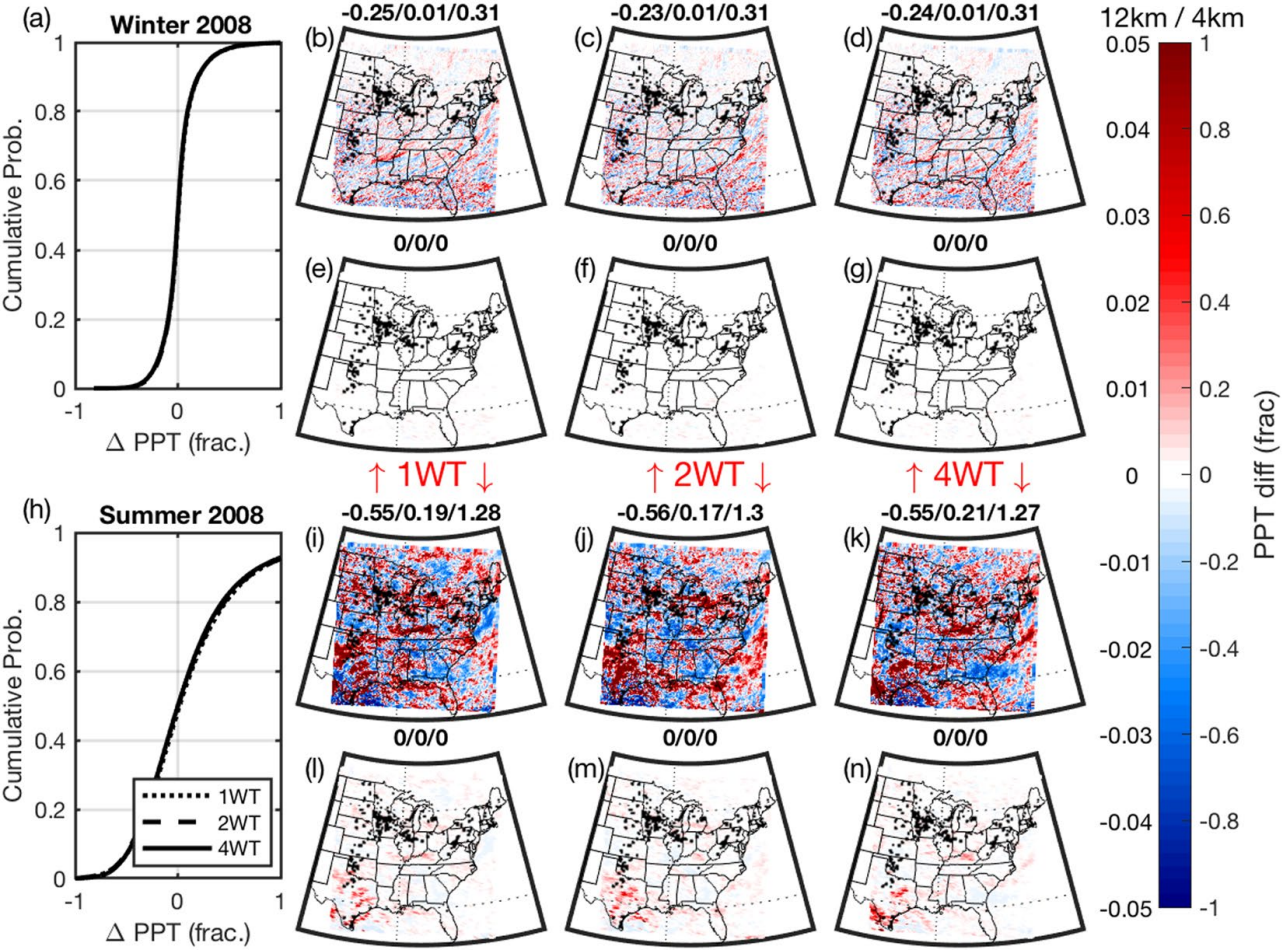
Impact of the presence of WT on precipitation (PPT) as a function of spatial aggregation in winter (DJF) and summer (JJA). (**a,h**) Cumulative probability of grid cell seasonal total PPT (XWT minus noWT) expressed as a fraction of precipitation receipt in the noWT simulation during winter (DJF) and summer (JJA) of 2008 for the three WT scenarios; 1 WT, 2 WT and 4 WT. (**b**), (**c**) and (**d**) Spatial fields of these normalized differences. **(i**–**k**) as for (**b**–**d**) but for summer. The panels (b,i) depict results for 1 WT, (**c**,**j**) are for 2 WT and (**d**,**k**) are for 4 WT. The lower panels (e–g) and (l–n) show results for analyses in which the precipitation perturbations are spatially averaged to 12 by 12 km grid cells. The color bar on the right show the scales for the output at 4 km (range; −1 to 1) and aggregated to 12 km (−0.05 to 0.05). Figure produced using MATLAB 2018a (https://www.mathworks.com).

A theoretical analysis conducted using an atmosphere-only Global Circulation Model configured with 8 vertical levels and a resolution of approx. 3.75° × 3.75° used an increase in drag coefficient (from 0.0024 to 0.0096) to simulate the effects of WT and found “installations of wind and solar farms covering the Sahara lead to a local temperature increase and more than a twofold precipitation increase, especially in the Sahel”^47^. Our high-resolution simulations of realistic WT deployments over the eastern USA indicate considerably more modest impacts on regional precipitation. Median grid cell differences in ΔPPT are <5 mm and even in grid cells containing WT the enhancement of summer PPT is <28 mm (cf. domain-wide seasonal average of 428–450 mm). This impact on seasonal PPT does not appear to scale with WT installed capacity (Fig. 6(c)), and in contrast to the study of a mega wind farm deployed in the central Great Plains^48^ the spatial patterns do not indicate specific enhancement of precipitation over the southeastern USA (Fig. 7). Large magnitude perturbations of seasonal total precipitation in individual 4 × 4 km grid cells are indicated during summer and fall. However, the effects are not present in winter or spring. They are spatially incoherent and are not robust to spatial averaging (Figs. 6(c)) and 7). When the pairwise differences in each 4 × 4 km grid cell are aggregated up to 12 × 12 km (i.e. perturbations are averaged over nine, 4 × 4 km grid cells) the results imply much smaller perturbations of PPT (Fig. 7). When remapped to 12 × 12 km fewer than 2% of grid cells exhibit a perturbation of >|1%| in seasonal precipitation in any season or WT scenario. Previous research^49,50^ has shown that while numerical models that employ grid-spacing of 1–4 km (in which convective is explicitly resolved) are ‘capable of producing more realistic simulations of larger convective entities….. greater realism does not necessarily mean more accurate forecasts’^50^. It has been further suggested that the skillful scale for these simulations under deeply convective conditions is likely tens of kilometers^50^, and that convection permitting simulations are sensitive to grid-scale noise and predictability limitations^51^. The inference from this previous research and the analysis of signal as a function of spatial aggregation is that local (grid-cell specific) PPT impacts in these perturbation experiments may reflect numerical noise^52,53^ rather than a systematic change in local or regional precipitation regime.

**Figure 8:**
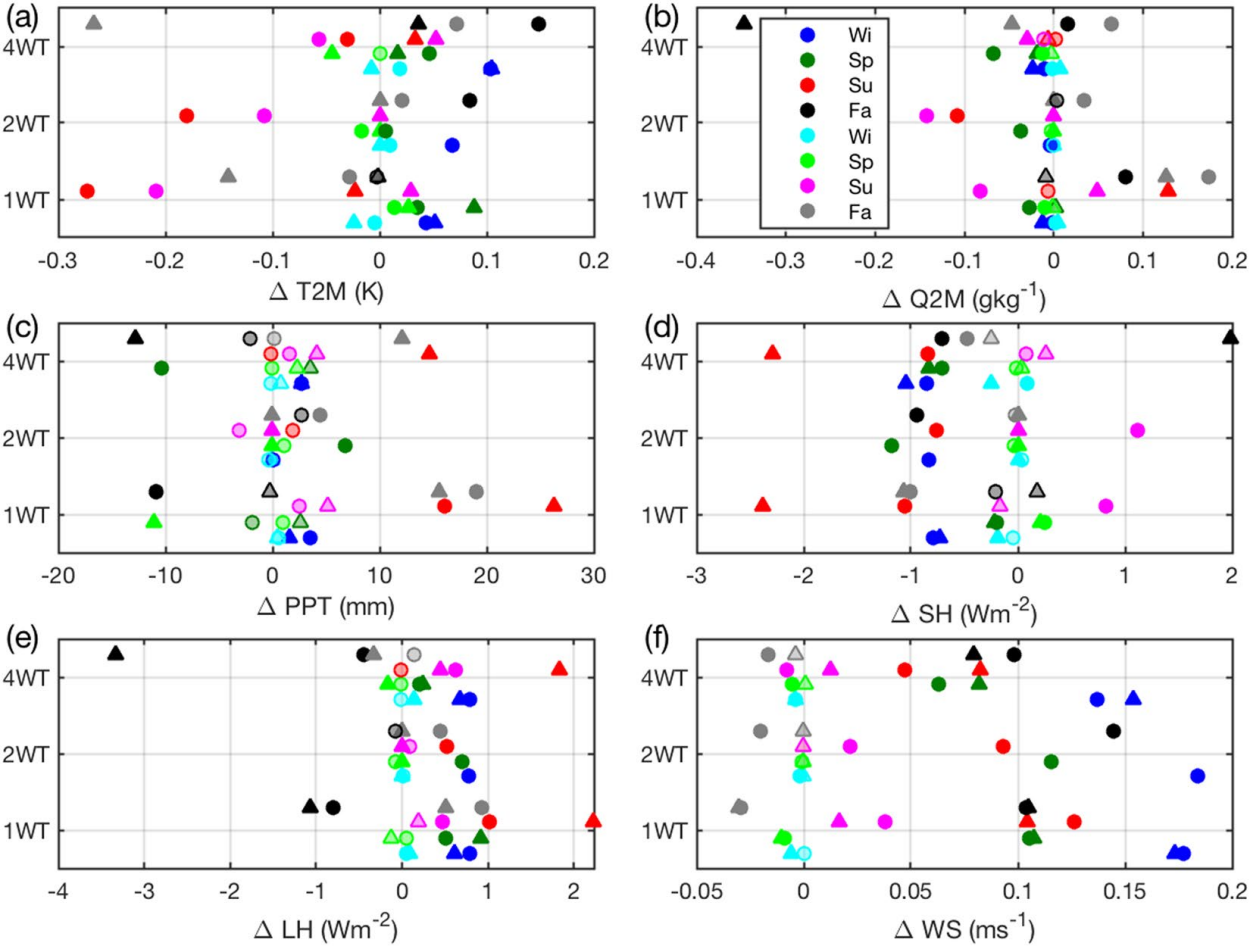
Median of grid cell mean of pairwise differences (XWT minus noWT) for all 1800 grid cells containing WT and an equal number of grid cells without WT for (**a**) air temperature at 2-m (T2M), (**b**) specific humidity at 2-m (Q2M), (**d**) sensible heat flux (SH), **(e**) latent heat flux (LH) and (**f**) and wind speed at 10-m (WS) for the three WT scenarios (1 WT, 2 WT, 4 WT). Panel (c) shows the difference in total seasonal precipitation (PPT). Filled circles denote results for 2008, while triangles show results for 2015/2016. Dominant colors show results for the WT grid cells while the lighter colors (i.e. cyan versus blue) show the 1800 randomly selected non-WT containing grid cells. Symbols that are filled with the edge color are associated with p-values < 0.05 for a sign test (these differences are deemed to be significantly different to zero). Test results with p > 0.05 are shown as partly transparent.

Median pairwise differences (XWT minus noWT) in each season for the 1800 grid cells containing WT and 1800 background grid cells (see Fig. 2(a) for locations, and discussion in Methods) were subject to a non-parametric sign test applied to test the null hypothesis that the samples of grid cell mean differences have zero median at a confidence level of 95%. According to this test, near-surface climate (T2M, Q2M, and the surface energy balance components; SH and LH) is impacted in grid cells containing WT in all seasons, both years and all WT scenarios. In addition to impacts on T2M, there is an increase in 10-m wind speed in WT grid cells (Fig. 8(f)) which along with the increased turbulence intensity impacts the surface energy balance. This effect is confined to grid cells containing WT in virtually all seasons and WT scenarios (Fig. 8(d,e)). In WT containing grid cells there is a significant decrease in SH fluxes during summer (median ΔSH; −0.5 to −2.5 Wm^−2^, depending on the scenario, relative to a domain-wide summertime mean ≈50 Wm^−2^) that is compensated for by increases in LH fluxes within those grid cells (median ΔLH; 0.3 to 2.2 Wm^−2^, summer <LH> ≈ 100 Wm^−2^) (cf. Fig. 7(d,e)). Conversely, even in the 4 WT scenario, the 1800 background grid cells exhibit median differences (4 WT – noWT) in T2M, Q2M, PPT, SH and LH that are close to zero and are generally not significantly different to zero (Fig. 8).

Analyses of the spatial decay of WT effects with increasing distance from grid cells containing WT indicate that, with the exception of T2M (Fig. 5(b)), for all downwind distances from WT grid cells there is an approximately equal probability of experiencing higher or lower values of all variables. Thus, even when WT are repowered to quadruple installed capacity the effect on near-surface climate appears to remain localized to the grid cells in which WT are deployed.

## Discussion

Actual evolution of the deployed WT fleet will inevitably be a mixture of expansion of WT deployment areas and repowering. The repowering scenarios used, where smaller capacity and dimension WT are replaced with larger models, were selected primarily to avoid competition for land. They represent a ‘worst case’ scenario for density of installed capacity and thus for potential saturation of the wind resource. There is indeed a small decrease in the overall efficiency of electrical power production as measured using annual mean gross capacity factors (CF) under the 4 WT scenario by repowering. This change in gross power production is approximately equal to the difference in the two years considered that arises from internal climate variability. It is possible that if the increase in installed capacity were realized by means other than repowering, quadrupling of electricity generation may be achieved by only quadrupling of installed capacity (i.e. that there would be no associated reduction in gross CF).

Quadrupling of installed capacity from the 2014 penetration levels increases total cumulative electricity generation over the eastern US from 131 to 474 TWh/yr for the climate of 2008 (La Niña year) and from 124 to 449 TWh/yr for the climate of 2015/2016 (El Niño year). Thus, quadrupling installed capacity over the eastern USA from 2014 levels solely by repowering increases gross electricity generation by a factor of ∼ 3.63. Projections from the US Energy Information Administration indicate net electricity supply to grid in 2030 of 4239 TWh (~7% increase from 2017/8)^54^. Assuming WT in the eastern USA continue to represent 48% of IC and electricity generation from WT, the 4 WT scenarios from repowering thus exceed the 20% from wind by 2030 goal.

WT modify near-surface climate in the grid cells where they are deployed, but the simulated impact on near-surface climate properties at the regional scale is modest even under scenarios of quadrupled WT installed capacity. Simulations presented indicate WT-induced impacts on climate variables at the local to regional scale are highly spatially variable, exhibit important inter-annual variability and decline in summer as larger capacity WT (and higher HH) are deployed. Both the current and increased WT capacity scenarios have modest impacts on near-surface climate variables that are largely confined to 4 by 4 km grid cells containing WT and to the summer season. For example, for the climate of 2008, the mean T2M perturbation across all the 1800 grid cells over the eastern US that contain WT is −0.27 K in summer and +0.04 K in winter in the 1 WT simulations. Comparable values for the 2 WT and 4 WT scenarios are; −0.18 K and +0.06 K and −0.03 K and +0.10 K.

High resolution simulations and analyses presented herein imply that even the local impacts on near-surface climate variables induced by the action of WT are modest compared to those induced by historical changes in land cover. Further, the regional impacts are smaller than the global temperature perturbation induced by use of coal to generate an equivalent amount of electricity. Assuming electricity generated from WT displaces generation from coal (30% of US electricity generation^8^), then the annual generation (of 474 and 449 TWh/yr) in the 4 WT scenario in 2008 and 2015/6 equates to carbon dioxide (CO_2_) emission reductions of 4.2 × 10^11^ and 4.0 × 10^11^ kg (using a net emission of 886 g-CO_2_ per kWh from coal versus wind power^55^). For a transient climate response to cumulative CO_2_ emissions (TCRE) of approx. 1.5 °C/1000 Gt-C (range of 0.8 to 2.5°C/1000 Gt-C)^56^, this means an avoided global warming from expansion of the WT installed capacity in the eastern USA of ~0.16–0.17 K.

## Methods

We conduct simulations using the Weather Research and Forecasting model (WRF, v3.8.1) for an outer domain resolved at 12 km and the inner domain discretized at 4 km. A 4 km resolution for d02 was selected for the following reasons: (i) To be at a scale referred to as “convection permitting” and thus avoid use of cumulus parameterizations while also avoiding ‘’Terra incognita” (defined by Wyngaard^57^ as a few hundred m) and the “gray zone” of turbulence (O(km)) at which assumptions employed in the other physics parameterizations (notably turbulence closure schemes) are no longer valid^58,59^. (ii) To be consistent with recommendations for realistic operation of wind farm parameterization (that the grid cell be of dimensions at least five times the WT rotor diameter)^60^ while also minimizing the ‘missing’ wake effect arising because all WT in a given grid cell experience the same inflow conditions and thus neglect WT interactions within a grid cell.

For all simulations the lateral boundary conditions are supplied every 6-hours from the ERA-Interim reanalysis data^61^. The NOAA Real Time Global sea surface temperature (RTG-SST) data set is used to provide initial SST and Great Lakes conditions and are updated every 24 hours.

Previous research has indicated strong teleconnections between near-surface and mid-tropospheric wind speeds over North America and internal modes of climate variability such as the phase of the El Niño-Southern Oscillation (ENSO)^26–28^. Given the importance of wind speed to wake generation from WT, WT electricity generation and downstream climates impacts may also exhibit important inter-annual variability^45^. Thus, simulations are presented for the calendar year 2008 to represent a moderate La-Niña and for March 2015-February 2016 to represent the positive phase (El Niño) conditions. Due to computational limitations the 2 WT scenario was not simulated for 2015/6.

Downstream impacts from WT are described using a wind farm parameterization designed for use with WRF^21,60^ and georeferenced WT types and specifications for deployments as of December 2014. The wind farm parameterization works such that every WT in a grid cell applies a (local) drag force (to act as a momentum sink) and turbulent kinetic energy (TKE) is added to all model vertical levels that intersect the turbine rotor. The drag applied and TKE introduced to the WRF TKE budget (see discussion in Supplemental Information) are determined by the incident wind speed and WT specifications (see Fig. 2(b) and Table 1).

Simulations presented herein consumed over 500,000 CPU hours and took over a calendar year to complete. They were all performed for 12-month continuous periods and multiple WT scenarios and employed a horizontal resolution of 4 km by 4 km and a vertical discretization of 41 levels. WRF uses sigma coordinates and thus the height of the vertical levels varies in time and space. For most of the simulation domain that equates to seven vertical layers below 250 m a.g.l. This is consistent with previous studies with WRF^22,23,62^, but is lower than the highest resolutions employed in studies over smaller simulation domains and/or shorter simulation periods^21,63^. A sensitivity analysis was performed to examine the impact of doubling the number of vertical levels in the lowest portion of the atmosphere by inserting layers at the mid-point between those in the 41 vertical layer simulation or quadrupling the number of horizontal grid cells by reducing the horizontal resolution to 2 km. This sensitivity analysis was conducted for a nested domain from ~103 to 83°W and ~33.5 to 49.5°N over the period 8–31 May 2008 with output analyzed for 15–31 May. These simulations are centered on Iowa because it is the state with highest WT density with, at the end of 2014, over 5.18 GW in ∼145,000 km^2^. Simulations were performed at (a) 4 km resolution with 41 vertical layers (as in the simulations presented for the entire eastern USA, Orig), (b) at 2 km horizontal resolution with 41 vertical levels (Enh. hor.) and (c) at 4 km resolution with double the number of vertical levels below 1 km (Enh. vert.) for 1 WT. The three time series of 10-minute cumulative power output from all WT in Iowa for all 10-minute periods during 15–31 May 2008 are highly correlated (r > 0.98), but the absolute magnitudes exhibit some differences. The mean and median ratio of total electrical power output for all 10-minute periods during 15–31 May 2008 expressed as Orig./Enh. hor. are 1.0007 (mean) and 0.9926 (median) and for Orig/Enh. vert. are 1.0035 and 0.9824, respectively. The results are consistent with previous research^21^, and indicate some sensitivity of wake effects, gross CF and power production to model vertical and horizontal resolution. In this sensitivity study the mean wind turbine array wake intensity is reduced and mean WT power production is enhanced when the number of vertical levels is doubled but exhibit little sensitivity to decreasing the horizontal resolution. However, the wake intensity was enhanced by increased vertical discretization in the study of a theoretical wind farm^21^. Hence, the magnitude and sign of the impact on gross CF from the vertical and horizontal resolution is likely to be modest (∼1%) and also to vary with the WT deployed, array layouts and prevailing meteorology.

As documented in the NameList provided in SI, the simulations presented herein were performed with TKE advection enabled to allow propagation of WT-induced TKE downstream from grid cells containing one or more WT (see SI Fig. 1). Recent work has illustrated that selection of this option tends to decrease TKE within a wind farm due to the influence from surrounding grid cells but, as expected, to increase it downstream as a manifestation of the whole wind farm wake^63^.

Our focus is on perturbation of the near-surface climate (i.e. expected atmospheric state) not instantaneous or short-term perturbations of the meteorology at the local and regional scale. We employ five different analyses to quantify local versus regional climate effects. First, we summarize spatial patterns of the mean pairwise perturbations of near-surface climate variables and examine them for spatial coherence. These mean perturbations are derived by computing pairwise (in time) differences in model output (XWT-noWT, where X = 1, 2 or 4) and using them to compute mean grid cell specific differences for each climatological season. The 5^th^ to 95^th^ percentile span and central tendency (50^th^ percentile) values of these differences across all d02 grid cells are used to evaluate the average domain-wide (regional scale) magnitude (and 90^th^ percentile range) of WT impacts. Differences (XWT - noWT) in each season are also computed pairwise for the 1800 grid cells containing WT and 1800 randomly selected land-based background grid cells that do not contain WT and are displaced by at least two grid cells from a WT. A non-parametric sign test is applied to test the null hypothesis that the sample of grid cell mean differences has a continuous distribution with zero median (two-tailed test, with significance assessed at p ≤ 0.05). This analysis thus examines the impact of WT on grid cells in which WT are located and also on an equal number of ‘background’ grid cells. The impact of WT on different parts of the T2M probability distribution, is evaluated by computing the mean difference (XWT - noWT) in the 1800 WT grid cells and the 1800 background grid cells as a function of each decile in the probability distribution. In this analysis, the 5^th^ to 95^th^ percentile values (at every 5^th^ percentile) of near-surface air temperature (T2M) in each WT grid cell is computed along with the difference XWT minus noWT at that percentile of T2M. The results are then averaged over all 1800 WT (or background) grid cells to compute the mean perturbation at that point on the probability distribution of T2M values. If, for example, the presence of WT tends to lead to local warming of nocturnal temperatures this will be manifest as a positive perturbation in the lowest percentiles of the T2M distribution. The mean grid cell differences in the near-surface climate variables are also explored in the context of distance from the closest WT. In this analysis the mean difference in each grid cell is conditionally sampled by the distance to the closest grid cell in which one or more WT is deployed. The probability that a grid cell at that distance exhibits a difference in excess of specified thresholds is then computed and presented as the probability that a given cell at that distance from a WT has a perturbation of greater than that magnitude. For example, for T2M the threshold is set at 0.2 K. The probability that a given cell at that distance from a WT has a T2M perturbation >+0.2 K is shown as positive, while the probability that a given cell at that distance from a WT exhibits cooling in excess of 0.2 K are shown as negative. Finally, the impact of WT operation on near-surface air temperatures is conducted as a function of hour of the day by season.

Further details of the simulations, wind turbine properties, scenario assumptions and justifications and evaluation of the base climate in the noWT simulation relative to *in situ* observations and a reanalysis product are presented in Supporting Information (SI).

## Supplementary information

Supplementary Information.

## Data Availability

Locations and types of all WT deployed in the continental USA as of December 2014 are available from https://eerscmap.usgs.gov/uswtdb/data/. ERA-interim output is available from http://apps.ecmwf.int/datasets/. The NOAA-NCEP Real Time Global sea surface temperature analyses are available from http://www.nco.ncep.noaa.gov/pmb/products/sst/. Output from MERRA-2 is available from; https://disc.sci.gsfc.nasa.gov/datasets?page=1&keywords=MERRA-2. Wind speeds for NWS ASOS stations are available from (ftp://ftp.ncdc.noaa.gov/pub/data/asos-fivemin/). WT power curves for all of the top eight most common WT types are available from http://www.wind-power-program.com/download.htm. Those and a range of other power curves for less commonly deployed WT are also available for purchase from www.thewindpower.net. Source code for WRF v3.8.1 is available from http://www2.mmm.ucar.edu/wrf/users/download/get_sources.html. Data presented in all figures can be obtained from the ZENODO repository (10.5281/zenodo.3595571).
