# Supplementary material for: 20% of US electricity from wind will have limited impacts on system efficiency and regional climate
Source: Sci Rep. 2020 Jan 17;10:541. doi: 10.1038/s41598-019-57371-1 (PMC6969061; doi:10.1038/s41598-019-57371-1)
Supplement: Supplementary file 1 — Supplementary Information. [file 41598_2019_57371_MOESM1_ESM.pdf]

Supporting Information for:

20% of US electricity from wind will have limited impacts on system efficiency and regional climate

S. C. Pryor[1*] (ORCID: 0000-0003-4847-3440). Email: sp2279@cornell.edu

R. J. Barthelmie[2] (ORCID: 0000-0003-0403-6046) . Email: rb737@cornell.edu

T.J. Shepherd[1] (ORCID: 0000-0001-8627-6419). Email: tjs346@cornell.edu

[1]Department of Earth and Atmospheric Sciences, Cornell University, Ithaca, New York 14853

[2]Sibley School of Mechanical and Aerospace Engineering, Cornell University, Ithaca, New York 14853

*Corresponding author: S.C. Pryor (sp2279@cornell.edu). Tel: 1-607-255-3376. Fax: 1-607-255-2106

Numerical simulations

Our work does not seek to characterize individual wind turbine wakes or to develop projections of future wind installed capacity project locations. Rather, the research objectives are to represent the cumulative impact from wind turbine arrays on system-wide electricity generation efficiency and near-surface regional climate over the eastern USA and to explore how those impacts may evolve under scenarios of increased installed capacity (IC).

The simulation domain, with an inner domain of 2540 km by 2540 km (resolved at 4 km by 4 km) permits an evaluation of the cumulative impact of actual deployed wind turbines at relatively high resolution but over an extensive area. This study is unique in the literature. No previous research has used such a highly resolved but spatially extensive simulation domain and simulated multiple years with real wind turbine (WT) characteristics for known WT locations

and scenarios of future installed capacities. All simulations were performed using WRF v3.8.1 compiled with the INTEL Fortran compiler on the NSERC Cori (Cray XC40) supercomputer. Based on an optimization experiment the simulations used 256 cores with 50% packing on individual nodes. These simulations consumed over 500,000 CPU hours and took over a year to complete.

The Fitch parameterization[1] of wind farm effects is employed in simulations presented herein, along with explicit details of each WT hub-height (HH), rotor dimeter (D), location, power and thrust curves (see Table 1 in the main document).

Physics schemes applied are (see NameList in the Appendix):

- Longwave radiation: Rapid radiative transfer model (RRTM)[2].
- Shortwave radiation: Dudhia[3]
- Microphysics: Eta (Ferrier)[4].
- Surface-layer physics: MM5 similarity scheme[5]
- Land surface physics: Noah land surface model[6].
- Planetary boundary layer: Mellor-Yamada-Nakanishi-Niino 2.5 (ref [7]). Note that the Fitch wind farm parameterization is designed to be used with this PBL scheme.
- Cumulus parameterization: Kain-Fritsch[8] is used in d01 while d02 is sufficiently highly resolved (4 km) that no cumulus scheme is applied.

Three-dimensional fields of the wind components and 10-m wind speeds analyzed herein are output every 10-minutes along with estimated power output in each grid cell. All other parameters are output once hourly.

Advection of turbulent kinetic energy (TKE) was enabled in the simulations presented herein. In the Fitch wind farm parameterization the fraction of kinetic energy extracted by the WT that is

not converted into electrical power is assumed to represent TKE that is added to the atmosphere from the action of WT and is added as a term in in the TKE equation in the RANS formulation[1]. This additional TKE scales with the wind speed cubed and tends to exceed that from shear production. Allowing TKE advection permits propagation of TKE from adjacent grid cells into those containing WT and TKE produced from WT to be transported into adjacent cells that do not contain WT, resulting in proper characterization of the enhancement of TKE in the 'wake' from wind farms (see example in SI Figure 1 and also figures in Fitch[1] that illustrate the modification of TKE by the action of wind farms). Consistent with *a priori* expectations recent work for an offshore wind farm has suggested use of TKE advection led to a negative bias in grid cells containing WT but improves the degree of agreement with observations downstream of an offshore wind farm[9].

Wind turbine characteristics and power production

Locations and types of all WT deployed in the continental USA as of December 2014 were obtained from https://eerscmap.usgs.gov/arcgis/rest/services/wind/wTurbinesWMDyn/MapServer and used in the 1WT simulations. The hub-heights (HH) and rotor diameters (D), rotor aerodynamics as described using WT power and thrust curves were then obtained for each WT type for which explicit information is available (see Table 1). Fewer than 2% of the > 18,200 WT are not one of the 28 most common WT types for which explicit power and thrust curves were applied and thus had to be allocated to one of two synthetic WT characteristic definitions. An example of an average thrust curve used when turbine specific information was not available is given in Table S1.

In addition to simulations conducted without WT and with the WT installed as of the end of 2014 (1WT), two repowering scenarios are also simulated. In these scenarios older, smaller capacity WT are replaced by larger, high rated capacity (RC) machines. The specifications of the WT used in the repowering scenarios are consistent with the market trend tendency towards higher RC and larger D with more modest increases in WT HH (Figure 1b). In principle moving to higher WT HH and D should yield power production gains due to the increase in swept area and typical increase of wind speeds with height (although complex vertical profiles can be observed particularly in heterogeneous terrain and landscapes)[10,11]. The choice of the specific WT used in the repowering scenarios was informed by three considerations: (a) Availability of WT power and thrust curves. Use of the wind farm parametrization in WRF requires each individual turbine be specified in terms of a geolocation, HH, D , power and thrust curves. The power and thrust curves of commercial WT are confidential. Thus the repowering scenarios employ WT models that either the authors were able to obtain under an NDA (the Vestas 3 MW WT) or that have been developed for use in the research community (the NREL 5.2 MW, and LW demonstration machine, see Table 1 of the main document)[12,13]. (b) The WT be consistent with trends in WT dimensions (i.e. are not beyond those of WT being considered for deployment). Over the last decade (2010-2018) the hub-height of the average utility-scale turbine in the USA increased from 80 to 88 m while the rotor diameter increased from 84 to 116 m (Figure 1 main text)[14]. Thus, the recent evolution in physical dimensions has tended to be manifest as modest increases in HH, with relatively larger increases in D and IC. Forty-seven percent of new installations during 2017 had a hub-height of 80 m, and that continued to be the most common HH in 2018 (ref. [14]). The new 5.3 MW onshore wind turbine from General Electric (Cypress), launched in September 2018, is offered with a hub-height of either 101 or 120

m. Thus, the WT types used for the scenarios (see Table 1) are consistent with larger-capacity machines that are just being released to the market. (c) That they are unlikely to result in declines in social acceptance due to very much larger hub-heights and/or limitations imposed by set-back regulations[15]. They are further informed by knowledge that Federal Aviation Authority approval process requirements are greatly enhanced for WT that have rotor tip heights above 499 ft (152 m). However, this is a very dynamic industry and it is possible that large increments in WT dimensions (HH or D) or IC will be realized even in the near-term.

As described in the main text, a threshold for maximum installed capacity density is applied to the repowering scenario used to quadruple installed capacity. That threshold is set at 16.25 $MWkm^{-2}$ (i.e. 260 MW in a 16 km^2 grid cell). This threshold is based on reported installed capacities from onshore and offshore wind farms[16,17]. There is considerable variability in reported installed capacity densities largely due to variations in the method employed to compute the area occupied by a wind turbine array. Onshore developments typically have very irregular layouts, which leads to low bias in installed capacity densities if a rhombus is used to determine the denominator (area). In 2009 two major reports were released, one focused on the USA and one from Europe. The US study found that 'Excluding the outliers, the reported data represents a capacity density range of 1.0 to 11.2 MW/km^2' (ref. [18]). The study focused on Europe found mean installed power density onshore and offshore of 8 and 10 $MWkm^{-2}$, respectively (ref. [19]). Installed capacity in the USA has since trebled (Figure 1) from 35 GW to > 100 GW, while the IC in Europe rose from 75 GW in 2009 to > 189 GW in 2018. New research has suggested these estimates from 2009 are biased low due to incorrect estimation of the land area used[16]. For the doubling installed capacity scenario (2WT) no grid cell exceeds the mean value for onshore wind farms in Europe as reported in 2009 (of 8 $MWkm^{-2}$). Even for the 4WT scenario the majority of

grid cells have an IC density < 8 MWkm^{-2}, and only 15 of the 455,625 grid cells have an IC density of 12.5 to 16 MWkm^{-2}.

As stated in the main text it is unlikely that all increases in WT IC will be realized through repowering. However, these scenarios are fully transparent in terms of the underlying assumptions and are used in the current research for four primary reasons: (a) They avoid competition for land and thus avoid assumptions regarding availability of additional land for WT developments[20]. (b) They are highly conservative of the potential to achieve 20% electricity from wind because they represent a 'worst case' scenario for IC density and thus the potential saturation of the wind resource[21]. (c) They ensure new capacity is added where there are current connections to the electricity distribution grid[22,23]. (d) They reflect an important trend in the industry. Both the Global Wind Energy Council[24] and the IEA[25] include " substantial and increased repowering" into their growth scenarios, and indicate repowering "becoming a significant factor after 2025" (ref. [24]). Tax incentives are being offered to enable repowering. A total of 3.6 GW of repowering projects were completed in 2017 and 2018 (ref [14]), in May 2019 GE announced it had repowered over 4 GW of its US fleet, and 30% of US wind farms are expected to undergo repowering by the end of 2020 (ref [26]).

Baseline climate from WRF simulations

The Weather Research Forecasting (WRF) model has been widely applied and evaluated[27-29] and there is an expectation that when applied within reanalysis-derived lateral boundary conditions the simulations will exhibit high fidelity[30]. Accordingly, near-surface (2-m a.g.l.) air temperature (T2M) and specific humidity (Q2M) and precipitation (PPT) from the 4 km WRF simulations without wind turbines (noWT) for both 2008 and 2015/2016 exhibit similarity with output from the Modern-Era Retrospective Analysis for Research and Application, Version 2 (MERRA-2)

reanalysis data set[31,32] (cf. Figure S2 and S3 with S4). The WRF simulations and independent reanalysis data for these two years also exhibit a similar response in near-surface climates over eastern North America to ENSO phase[33]. Spatially averaged specific humidity (Q2M) and total precipitation (PPT) over d02 were 6% lower during 2008 (negative phase ENSO) than 2015/2016 (positive phase) in independent MERRA-2 reanalysis output and 7% lower in the WRF simulations.

Evaluation of numerically simulated wind fields relative to in situ observations and/or gridded output at different resolutions is complex[28]. Nevertheless, an evaluation of near-surface wind speeds (at 10-m a.gl.) from the noWT WRF simulations was conducted relative to sonic anemometers at 293 National Weather Service Automated Surface Observing Stations (ASOS). While these observations are subject to recording biases (e.g. rounding up to the nearest knot)[34], mean annual wind speeds for 2008 from the 4 km and 12 km domains indicate mean ratios with observational values of 0.71 and 0.70 respectively, and spatial pearson correlation coefficients (r) with the observed values of 0.67 and 0.57 respectively. Thus, d02 wind speeds show increased similarity with observations over WRF output at 12 km providing an additional justification for the high resolution of the simulations presented herein. Nevertheless, consistent with previous research[35], there is a positive bias in wind speeds at 10-m from the WRF simulations. For comparison, the spatially averaged mean ratio of mean wind speeds from model output at 50 km and observations at 10 m a.gl. over Europe is 0.78 (ref [35]). This bias likely derives from unresolved topographic drag and that point observations may not be representative of grid cell mean conditions[36]. The degree of positive bias relative to in situ (point) observations generally declines with height as the influence of surface inhomogeneities decline[28], but nevertheless

represents a source of uncertainty in regional analyses of wind resources[37] and WT perturbations of flow and thermodynamic properties.

APPENDIX: WRF NAMELIST

```
&time_control
 run_days                    =  31,
 run_hours                   =  0,
 run_minutes                 =  0,
 run_seconds                 =  0,
 start_year                  =  2008,  2008,
 start_month                 =  08,    08,
 start_day                   =  01,    01,
 start_hour                  =  00,    00,
 start_minute                =  00,    00,
 start_second                =  00,    00,
 end_year                    =  2008,  2008,
 end_month                   =  09,    09,
 end_day                     =  01,    01,
 end_hour                    =  00,    00,
 end_minute                  =  00,    00,
 end_second                  =  00,    00,
 interval_seconds            =  21600
 input_from_file             =  .true.,.true.,
 history_interval            =  10,  10,
 frames_per_outfile          =  1,  1,
 history_outname             =
"/global/cscratch1/sd/tshep/WRF/WT_RUNS/WRFV3/2008_simulation_WT/files/wrfout/wrfout
_d<domain>_<date>"
 restart                     =  .true.,
 restart_interval            =  11160,
 override_restart_timers     =  .true.,
 io_form_history             =  11
 io_form_restart             =  2
 io_form_input               =  2
 io_form_boundary            =  11
 io_form_auxinput2           =  11
 io_form_auxhist2            =  11
 debug_level                 =  10
 nocolons                    =  .true.,
 auxinput4_inname            =  "wrflowinp_d<domain>",
 auxinput4_interval          =  1440,  1440,
 io_form_auxinput4           =  2,
 auxinput1_inname            =
"/global/cscratch1/sd/tshep/WPS_output/2008/met_em.d<domain>.<date>"
```

```
 iofields_filename                    =  "my_file_d01.txt", "my_file_d02.txt"
 ignore_iofields_warning              =  .true.,
 auxhist24_outname                    =
"/global/cscratch1/sd/tshep/WRF/WT_RUNS/WRFV3/2008_simulation_WT/files/wrfout/auxhis
t24_d<domain>_<date>"
 auxhist24_interval                   =  1440,  1440,
 frames_per_auxhist24                          =  1,  1,
 io_form_auxhist24                    =  11
 auxhist6_outname                  =
"/global/cscratch1/sd/tshep/WRF/WT_RUNS/WRFV3/2008_simulation_WT/files/wrfout/auxhis
t6_d<domain>_<date>"
 auxhist6_interval                 =  360,  360,
 frames_per_auxhist6                  =  1,  1,
 io_form_auxhist6                   =  11
 auxhist3_outname                  =
"/global/cscratch1/sd/tshep/WRF/WT_RUNS/WRFV3/2008_simulation_WT/files/wrfout/auxhis
t3_d<domain>_<date>"
 auxhist3_interval                 =  180,  180,
 frames_per_auxhist3                  =  1, 1,
 io_form_auxhist3                   =  11
 auxhist1_outname                  =
"/global/cscratch1/sd/tshep/WRF/WT_RUNS/WRFV3/2008_simulation_WT/files/wrfout/auxhis
t1_d<domain>_<date>"
 auxhist1_interval                 =  60,  60,
 frames_per_auxhist1                  =  1,  1,
 io_form_auxhist1                   =  11
/

 &domains
 time_step                          = 72,
 time_step_fract_num                  = 0,
 time_step_fract_den                  = 1,
 max_dom                            = 2,
 e_we                             = 320,    676,
 e_sn                             = 320,    676,
 e_vert                           = 41,    41,
 p_top_requested                    = 5000,
 num_metgrid_levels                   = 61,
 num_metgrid_soil_levels              = 4,
 dx                               = 12000, 4000,
 dy                               = 12000, 4000,
 grid_id                          = 1,     2,
 parent_id                          = 1,     1,
 i_parent_start                     = 1,     70,
 j_parent_start                     = 1,     35,
 parent_grid_ratio                    = 1,     3,
```

```
parent_time_step_ratio            = 1,     3,
feedback                          = 0,
max_ts_locs                       = 29,
eta_levels                        = 1.0000 , 0.9958 , 0.9916 , 0.9874 , 0.9832 ,
                                    0.9790 , 0.9749 , 0.9707 , 0.9661 , 0.9609 ,
                                    0.9549 , 0.9480 , 0.9398 , 0.9303 , 0.9189 ,
                                    0.9054 , 0.8894 , 0.8704 , 0.8481 , 0.8221 ,
                                    0.7922 , 0.7583 , 0.7205 , 0.6791 , 0.6346 ,
                                    0.5877 , 0.5393 , 0.4900 , 0.4407 , 0.3922 ,
                                    0.3450 , 0.2996 , 0.2564 , 0.2156 , 0.1773 ,
                                    0.1417 , 0.1086 , 0.0755 , 0.0475 , 0.0224 ,
                                    0.0000,
/

&physics
mp_physics                        = 5,     5,
ra_lw_physics                     = 1,     1,
ra_sw_physics                     = 1,     1,
radt                              = 15,    15,
sf_sfclay_physics                 = 1,     1,
sf_surface_physics                = 2,     2,
bl_pbl_physics                    = 5,     5,
bldt                              = 0,     0,
cu_physics                        = 1,     0,
cudt                              = 5,
isfflx                            = 1,
ifsnow                            = 1,
icloud                            = 1,
surface_input_source              = 3,
num_soil_layers                   = 4,
num_land_cat                      = 21,
sf_urban_physics                  = 0,     0,
bl_mynn_tkebudget                 = 1,
bl_mynn_tkeadvect                 = .true.,
rdmaxalb                          = .false.,
sst_update                        = 1,
tmn_update                        = 1,
usemonalb                         = .true.,
lagday                            = 150,
sst_skin                          = 1,
slope_rad                         = 1,
prec_acc_dt                       = 60.,
fractional_seaice                 = 1,
seaice_threshold                  = 0.,
windfarm_opt                      = 1, 1,
windfarm_ij                       = 0,
```

```
/

&noah_mp
dveg                = 4,
opt_crs             = 1,
opt_btr             = 2,
opt_run             = 3,
opt_sfc             = 1,
opt_frz             = 1,
opt_inf             = 1,
opt_rad             = 3,
opt_alb             = 2,
opt_snf             = 4,
opt_tbot            = 1,
opt_stc             = 3,
/

&dynamics
w_damping           = 1,
diff_opt            = 1,      1,
km_opt              = 4,      4,
diff_6th_opt        = 0,      0,
diff_6th_factor     = 0.12,   0.12,
base_temp           = 290.
damp_opt            = 0,
zdamp               = 5000.,  5000.,
dampcoef            = 0.01,    0.01,
khdif               = 0,      0,
kvdif               = 0,      0,
non_hydrostatic     = .true., .true.,
/

&bdy_control
spec_bdy_width      = 5,
spec_zone           = 1,
relax_zone          = 4,
spec_exp            = 0.13
specified           = .true., .false.,
nested              = .false., .true.,
/

&grib2
/

&namelist_quilt
nio_tasks_per_group = 0,
```

```
nio_groups = 1,
/
```

Table S1. Average thrust coefficients (c_t) by wind speed used for WT for which specific thrust coefficients are not available.

Wind speed (ms^{-1})	Average c_t
4	0.99
5	0.91
6	0.88
7	0.86
8	0.84
9	0.78
10	0.69
11	0.58
12	0.46
13	0.36
14	0.29
15	0.23
16	0.20
17	0.17
18	0.15
19	0.12
20	0.11
21	0.11
22	0.10
23	0.10
24	0.09
25	0.09

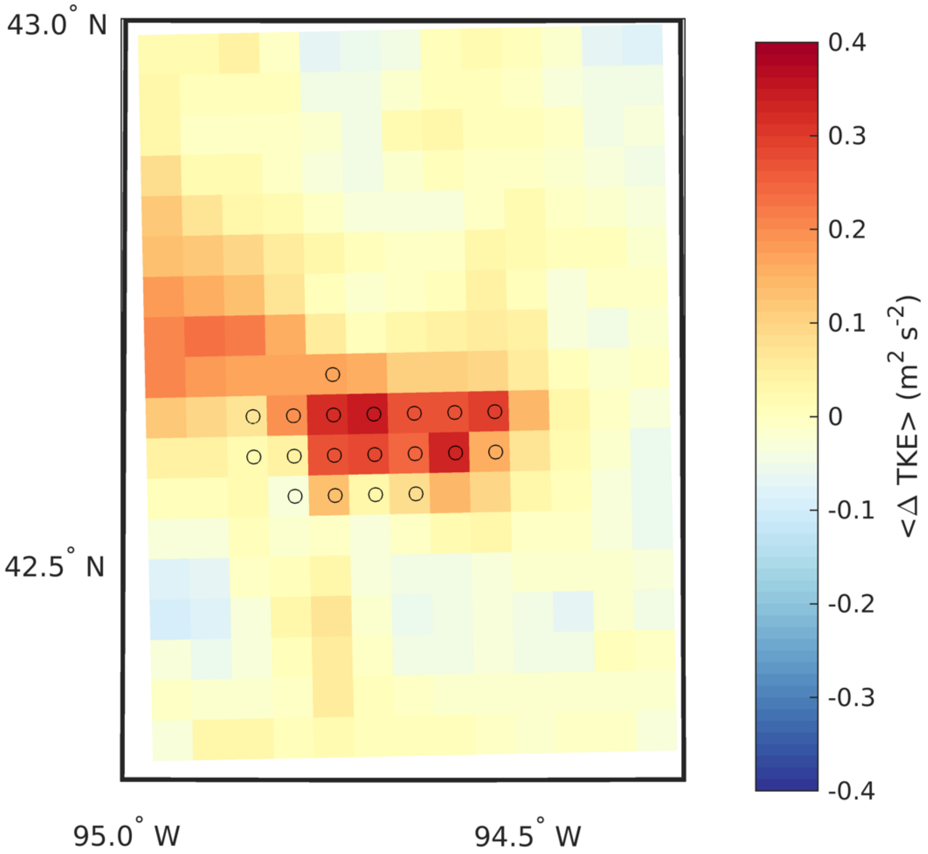

Figure S1 | Mean difference in TKE close to WT hub-height (TKE in 1WT minus noWT) over a cluster of wind turbines (WT) in Iowa computed from 10-minute output from the 1WT and noWT simulations of August 2008. Grid cells containing one or more WT are shown by the black circles.

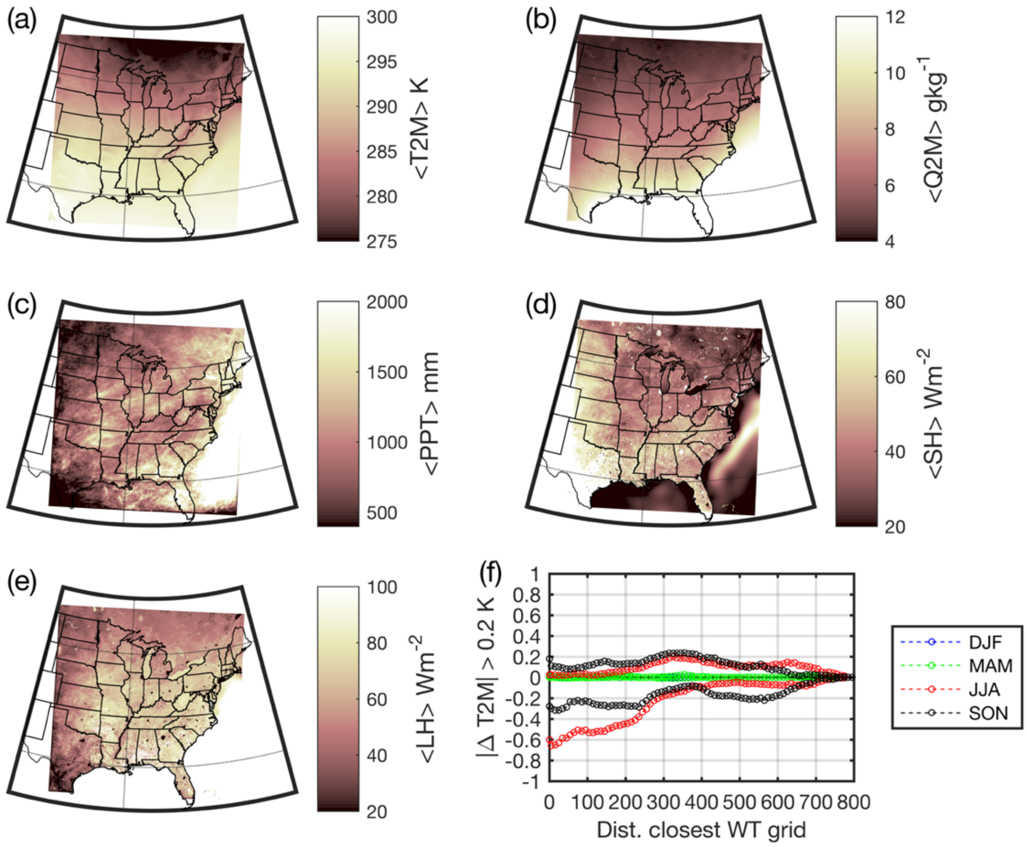

Figure 2 | Mean spatial patterns of (a) air temperature at 2-m (T2M) in K, (b) specific humidity at 2-m (Q2M) in gkg^{-1}, (c) annual total precipitation (mm), and mean (d) sensible heat flux (SH) in Wm^{-2}, (e) latent heat flux (LH) in Wm^{-2} in the noWT simulation for 2008. (f) Probability of a mean T2M perturbation > 0.2 K in the 4WT for each climatological season of 2008 for 4WT minus noWT as a function of distance (in km) from the closest grid cell containing WT. The y-axis on (f) is scaled such that the probability that a given cell at that distance from a WT has a perturbation of +0.2 K is shown as positive, while the probability that a given cell at that distance from a WT exhibits cooling in excess of 0.2 K are shown as negative. For example; in summer for a grid cell 100 km from the closest WT has a 50% chance of showing a cooling of > 0.2 K, and a 5% chance of showing warming of > 0.2 K. Very high SH

and low LH fluxes shown in (d) and (e) are for grid cells with urban land cover. Figure produced using MATLAB 2018a (https://www.mathworks.com).

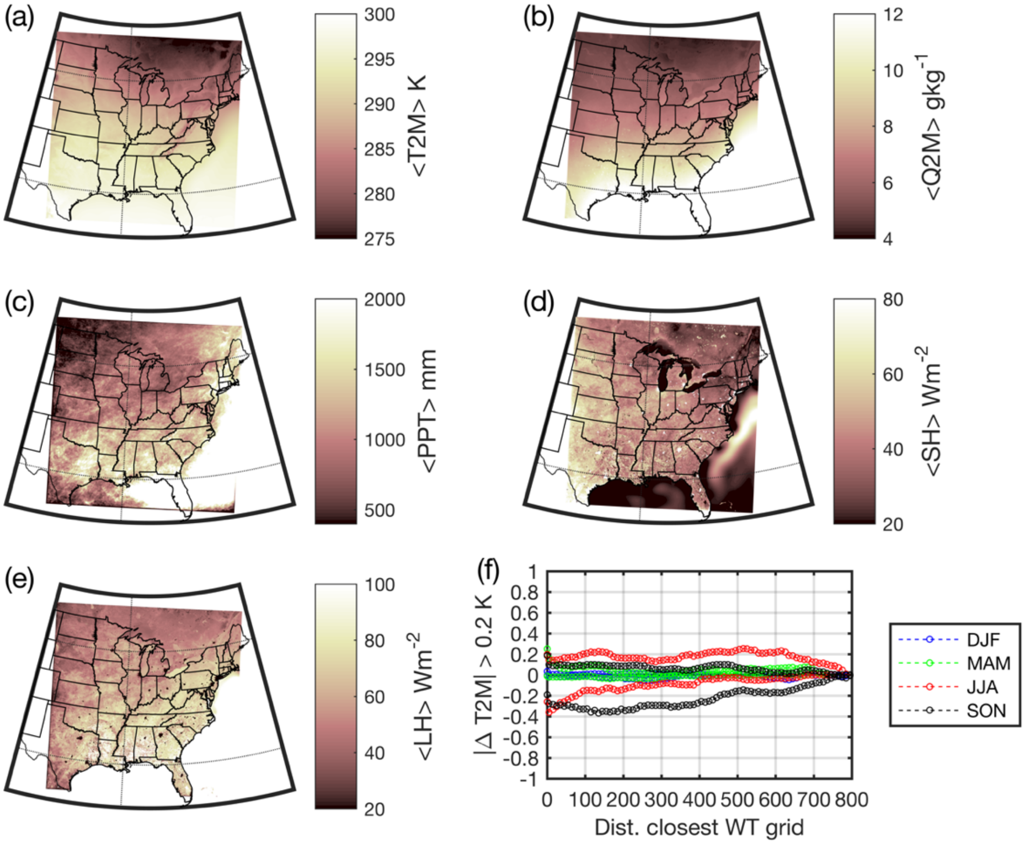

Figure S3 | Mean spatial patterns of (a) air temperature at 2-m (T2M) in K, (b) specific humidity at 2-m (Q2M) in gkg^{-1}, (c) annual total precipitation (mm), and mean (d) sensible heat flux (SH) in Wm^{-2}, (e) latent heat flux (LH) in Wm^{-2} in the noWT simulation for 2015/2016. (f) Probability of a mean T2M perturbation > 0.2 K in the 4WT for each climatological season of 2015/6 for 4WT minus noWT as a function of distance (in km) from the closest grid cell containing WT. The y-axis on (f) is scaled such that the probability that a given cell at that distance from a WT has a perturbation of +0.2 K is shown as positive, while the probability that a given cell at that distance from a WT exhibits cooling in excess of 0.2 K are shown as negative. For example; in summer for a grid cell 100 km from the closest WT

has a 50% chance of showing a cooling of > 0.2 K, and a 5% chance of showing warming of > 0.2 K. Figure produced using MATLAB 2018a (https://www.mathworks.com).

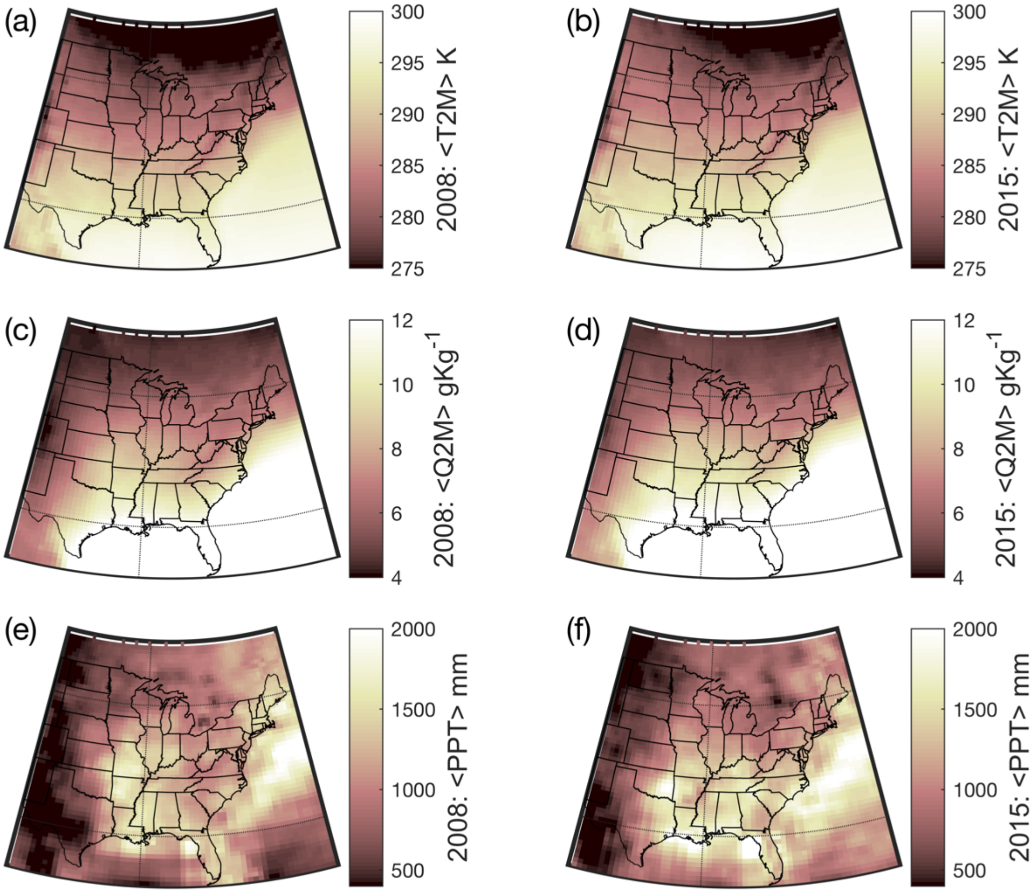

Figure S4 | Mean spatial patterns of (a) and (b) air temperature at 2-m (T2M) in K in 2008 and 2015/2016, (c) and (d) specific humidity at 2-m (Q2M) in gkg^{-1} in 2008 and 2015/2016 and (e) and (f) annual total precipitation (mm) in 2008 and 2015/2016 from the MERRA2 reanalysis. Values show in these panels can be compared with those from the WRF 4 km simulations as shown in Figure S2 and S3. Figure produced using MATLAB 2018a (https://www.mathworks.com).

References

1 Fitch, A. C. *et al.* Local and mesoscale impacts of wind farms as parameterized in a mesoscale NWP model. *Monthly Weather Review* **140**, 3017-3038, doi:https://doi:10.1175/mwr-d-11-00352.1 (2012).

2 Mlawer, E. J., Taubman, S. J., Brown, P. D., Iacono, M. J. & Clough, S. A. Radiative transfer for inhomogeneous atmospheres: RRTM, a validated correlated-k model for the longwave. *Journal of Geophysical Research: Atmospheres* **102**, 16663-16682 (1997).

3 Dudhia, J. Numerical study of convection observed during the winter monsoon experiment using a mesoscale two-dimensional model. *Journal of the Atmospheric Sciences* **46**, 3077-3107 (1989).

4 Ferrier, B. S. *et al.* Implementation of a new grid-scale cloud and precipitation scheme in the NCEP Eta model. *15th Conf. on Numerical Weather Prediction*, 280-283 (2002).

5 Beljaars, A. The parametrization of surface fluxes in large-scale models under free convection. *Quarterly Journal of the Royal Meteorological Society* **121**, 255-270 (1995).

6 Tewari, M. *et al.* Implementation and verification of the unified NOAH land surface model in the WRF model. *20th Conference on Weather Analysis and Forecasting/16th Conference on Numerical Weather Prediction* **1115**, 6pp (2004).

7 Nakanishi, M. & Niino, H. An improved Mellor–Yamada level-3 model: Its numerical stability and application to a regional prediction of advection fog. *Boundary-Layer Meteorology* **119**, 397-407 (2006).

8 Kain, J. S. The Kain–Fritsch convective parameterization: an update. *Journal of Applied Meteorology* **43**, 170-181 (2004).

9 Siedersleben, S. K. *et al.* Observed and simulated turbulent kinetic energy (WRF 3.8.1) overlarge offshore wind farms. *Geoscientific Model Development Discussions* **In review**, 1-29, doi:10.5194/gmd-2019-100 (2019).

10 Sathe, A., Gryning, S.-E. & Peña, A. Comparison of the atmospheric stability and wind profiles at two wind farm sites over a long marine fetch in the North Sea. *Wind Energy* **14(6)**, 767-780, doi:10.1002/we.456 (2011).

11 Peña, A., Gryning, S. E. & Mann, J. On the length-scale of the wind profile. *Quarterly Journal of the Royal Meteorological Society* **136**, 2119-2131 (2010).

12 Desmond, C., Murphy, J., Blonk, L. & Haans, W. Description of an 8 MW reference wind turbine. *Journal of Physics: Conference Series* **753**, 092013 (2016).

13 Jonkman, J., Butterfield, S., Musial, W. & Scott, G. Definition of a 5-MW Reference Wind Turbine for Offshore System Development Technical Report NREL/TP-500-38060. 75 (https://www.nrel.gov/docs/fy09osti/38060.pdf accessed 9 January 2019, 2009).

14 American Wind Energy Association. AWEA: Year End 2018: Market Report. 172 pp (Available for purchase from: https://www.awea.org/resources/publications-and-reports/market-reports/2018-u-s-wind-industry-market-reports, 2019).

15 Meyerhoff, J., Ohl, C. & Hartje, V. Landscape externalities from onshore wind power. *Energy Policy* **38**, 82-92 (2010).

16 Enevoldsen, P. & Jacobson, M. Z. Data investigation of installed and output power densities of onshore and offshore wind turbines worldwide. *Wind Energy* **In review** (2019).

17 NYSERDA. Analysis of Turbine Layouts and Spacing Between Wind Farms for Potential New York State Offshore Wind Development. 76 (Report Number 18-20, New

York State Energy Research and Development Authority, Albany, NY, Available from: https://www.nyserda.ny.gov/About/Publications/Offshore-Wind-Plans-for-New-York-State, 2018).

18 Denholm, P., Hand, M., Jackson, M. & Ong, S. Land use requirements of modern wind power plants in the United States. (National Renewable Energy Lab.(NREL), Golden, CO (United States). Available from: https://www.osti.gov/biblio/964608, 2009).

19 Swart, R. *et al.* Europe's onshore and offshore wind energy potential: An assessment of environmental and economic constraints. Report No. 9292130005, 90 (European Environment Agency, Technical report No 6/2009. Available from: https://www.eea.europa.eu/publications/europes-onshore-and-offshore-wind-energy-potential, Copenhagen, Denmark, 2009).

20 Maly, C. in *Renewable Energy Law in the EU: Legal Perspectives on Bottom-Up Approaches* (eds M. Peeters & T. Schomerus) 210-231 (Edward Elgar, Cheltenham, UK, 2014).

21 Miller, L. M. *et al.* Two methods for estimating limits to large-scale wind power generation. *Proceedings of the National Academy of Sciences* **112**, 11169-11174, doi:10.1073/pnas.1408251112 (2015).

22 Hitaj, C. Wind power development in the United States. *Journal of Environmental Economics and Management* **65**, 394-410 (2013).

23 Veers, P. *et al.* Grand challenges in the science of wind energy. *Science* **366**, eaau2027, doi:doi: 10.1126/science.aau2027 (2019).

24 Global Wind Energy Council. Global wind energy outlook 2016. 44 (Available from: http://files.gwec.net/files/GlobalWindEnergyOutlook2016. Brussels, Belgium, 2016).

25 International Energy Agency, I. Renewables 2019: Analysis and forecast to 2024. (IEA, France. ISBN 978-92-64-36998-6, Available from: https://www.iea.org/renewables2019/. 2019).

26 Dvorak, P. in *Windpower Engineering and Development* (Available at: https://www.windpowerengineering.com/projects/repowering/look-repowering-older-wind-farms-2020/ 2018).

27 Pryor, S. C., Barthelmie, R. J. & Shepherd, T. The influence of real-world wind turbine deployments on regional climate. *Journal of Geophysical Research: Atmospheres* **123**, 5804-5826 (2018).

28 Draxl, C., Hahmann, A. N., Peña, A. & Giebel, G. Evaluating winds and vertical wind shear from Weather Research and Forecasting model forecasts using seven planetary boundary layer schemes. *Wind Energy* **17**, 39-55 (2014).

29 Sun, X. *et al.* An evaluation of dynamical downscaling of Central Plains summer precipitation using a WRF-based regional climate model at a convection-permitting 4 km resolution. *Journal of Geophysical Research: Atmospheres* **121**, 13801-13825, doi:13810.11002/12016JD024796 (2016).

30 Castro, C. L., Pielke, R. A. & Leoncini, G. Dynamical downscaling: Assessment of value retained and added using the regional atmospheric modeling system (RAMS). *Journal of Geophysical Research* **110**, D05108, doi: 05110.01029/02004jd004721 (2005).

31 Molod, A., Takacs, L., Suarez, M. & Bacmeister, J. Development of the GEOS-5 atmospheric general circulation model: evolution from MERRA to MERRA2. *Geoscientific Model Development* **8**, 1339-1356 (2015).

32 Reichle, R. H. *et al.* Assessment of MERRA-2 land surface hydrology estimates. *Journal of Climate* **30**, 2937-2960 (2017).
33 Ropelewski, C. F. & Halpert, M. S. North American precipitation and temperature patterns associated with the El Niño/Southern Oscillation (ENSO). *Monthly Weather Review* **114**, 2352-2362 (1986).
34 Letson, F., Pryor, S. C., Barthelmie, R. J. & Hu, W. Observed gust wind speeds in the coterminous United States, and their relationship to local and regional drivers. *Journal of Wind Engineering and Industrial Aerodynamics* **173**, 199-209 (2018).
35 Vautard, R. *et al.* Regional climate model simulations indicate limited climatic impacts by operational and planned European wind farms. *Nature Communications* **5**, doi: 10.1038/ncomms4196 (2014).
36 Jiménez, P. A. & Dudhia, J. Improving the representation of resolved and unresolved topographic effects on surface wind in the WRF model. *Journal of Applied Meteorology and Climatology* **51**, 300-316 (2012).
37 Badger, J., Frank, H., Hahmann, A. N. & Giebel, G. Wind-Climate Estimation Based on Mesoscale and Microscale Modeling: Statistical–Dynamical Downscaling for Wind Energy Applications. *Journal of Applied Meteorology and Climatology* **53**, 1901-1919 (2014).
